# Temperature effects on food supply and chick mortality in tree swallows (*Tachycineta bicolor*)

**DOI:** 10.1007/s00442-013-2605-z

**Published:** 2013-03-07

**Authors:** David W. Winkler, Miles K. Luo, Eldar Rakhimberdiev

**Affiliations:** 1 Department of Ecology and Evolutionary Biology and Museum of Vertebrates, Cornell University, Ithaca, NY 14853 USA; 2 Cornell Laboratory of Ornithology, 159 Sapsucker Woods Rd, Ithaca, NY 14850 USA; 3 Department of Vertebrate Zoology, Lomonosov Moscow State University, Moscow, Russia

**Keywords:** Brood survival, Cold snaps, Daily survival rates, Insect abundance, Passerine bird

## Abstract

Tree swallow (*Tachycineta bicolor*) breeding success in Ithaca, NY, USA, over the past quarter century has shown generally healthy fledgling production punctuated by years of high nestling mortality. This study tested the potential effects that temperature may have on the food supply and breeding success of swallows. Data from 17 years of daily insect samples were used to relate flying insect abundances to daily maximum temperatures and to define “cold snaps” as strings of consecutive days when the maximum temperatures did not exceed critical temperatures. The distributions of cold snaps and chick mortality events were investigated both through detailed reconstructions of the fates and fate dates of individual chicks, focused on the three breeding seasons of lowest fledging success, and with less detailed brood-level analyses of a larger 11-year dataset including years of more moderate mortality. Mark–recapture analyses of daily brood survival rate (DSR) reveal very strong support for the effects of cold temperatures on brood survival rates, and all the top models agree on a critical temperature of 18.5 °C for insect flight activity in Ithaca. The individual-level analyses, focused on years of higher mortality, favored a 3-day cold snap definition as the most predictive of DSR effects, whereas the larger-scale brood-level analyses revealed 1- and 2-day cold snaps as having the most significant effects on DSR. Regardless, all analyses reveal that, in an age of generally warmer climates, the largest effect of weather on swallow fledgling production is from cold temperatures.

**Electronic supplementary material:**

The online version of this article (doi:10.1007/s00442-013-2605-z) contains supplementary material, which is available to authorized users.

## Introduction

Two of the most fundamental linkages in animal ecology are the effects on reproductive success from variation in a species’ food supply and the weather. For many species of birds, the measurement of the food supply can be a significant empirical challenge (Haftorn 1956; Tinbergen 1960; Sherry 1984; Wiens 1984; Holmes and Schultz 1988). Although previous studies have shown that changing food supply often has an effect on energy expenditure and breeding success or chick growth rates (Brinkhof and Cavé 1997; Zanette et al. 2003; Both 2010; Barichello and Mossop 2011; te Marvelde et al. 2011), few studies have shown this relationship in passerines through direct measures of both fledgling production and food abundance. The availability of invertebrate prey and the profitability of avian foraging on them can be higher at higher temperatures (e.g., Avery and Krebs 1984; Arlettaz et al. 2012), but especially so for aerial insectivores (Veistola and Lehikoinen 1997). The problem of food measurement for these species that take flying insect prey from the air is made easier by the availability of suction samplers that produce representative samples of available prey at frequent intervals, but this guild of consumers has the parallel challenge that day-to-day fluctuations in air temperatures are associated with dramatic variations in food supply (Hails and Bryant 1979; Emlen et al. 1991; McCarty 1995). Flying insects can disappear from the air column not because they have died but merely because they cannot fly in cold temperatures (Taylor 1963; Dunn et al. 2011). Thus, aerial insectivores are distinctive in that they are vulnerable to physiological, in addition to demographic, responses in their prey (Bryant 1973).

**Fig. 1:**
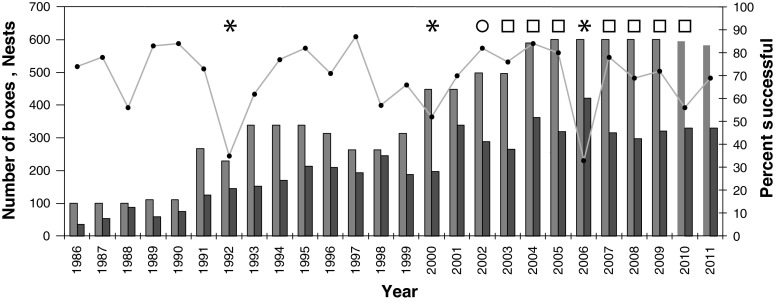
The number of available boxes (*green bar*) for nesting tree swallows (*Tachycineta bicolor*) around Ithaca, New York, from 1986 to 2011, the number of boxes used for nesting (*orange bar*), and the percentage of nests that successfully produced at least one fledgling (*solid line*). *Asterisks* indicate years of high brood mortality and *circle* for 1 year of normal brood survival for comparison, all used for individual chick-level mortality analyses in this paper. These years, plus years indicated with *squares*, were pooled for a brood-level analysis of cold snap effects (for details see text) (color figure online)

Tree swallows (*Tachycineta bicolor*) conveniently nest in boxes that allow for efficient monitoring of reproductive success, and food supply can be tracked through the use of suction samplers to collect flying insects (Macaulay et al. 1988). While typical years are characterized by more than 60 % of attempted nests successfully fledging at least one chick (McCarty and Winkler 1999a), approximately 1 out of every 3 years in upstate New York is characterized by high chick mortality rates with less than half of the nesting attempts able to produce at least one fledgling (Fig. 1). Using daily insect samples, daily weather records, and regular checks on chick survival, we directly test the link between air temperatures, insect availability, and chick survival. Note that cold weather, in addition to suppressing flying insect availability, likely increases metabolic demands of the chicks (e.g., McCarty 1995). We do not have long-term comparative data to measure the strength of this effect, and we concentrate on the effects of temperature evaluated solely on their measured effects on available insect numbers.

With cold temperatures suppressing aerial insect abundance, prolonged cold weather during the breeding season may further exacerbate the effects of low food availability. This, especially if coinciding with higher nutritional requirements, may result in higher offspring mortality rates and reduced breeding success. We here explore a distinctively detailed data set on the relation between ambient temperatures and available food, and using this relationship to define critical temperatures, investigate how the duration of periods of cold (“cold snaps”) interacts with temperature in its effects on patterns of chick and brood mortality. Using statistical methods adapted from mark-recapture studies, we test three linked hypotheses: that (1) flying insect availability in the air-column depends on air temperatures and that cold snaps have a significant impact on chick mortality depending on both their (2) temperatures and (3) durations.

## Materials and methods

Tree swallows readily accept artificial nest boxes, often breed at high local densities, and are remarkably tolerant of repeated disturbance from researchers (Jones 2003). Because of their wide geographic distribution (Winkler et al. 2011), they inhabit a broad range of ecosystems. Since the mid-1980s, tree swallows breeding in nest boxes have been studied throughout Tompkins County, around Ithaca, New York, USA.

To better understand the possible effects of temperature on swallow breeding success, flying insect abundances, and temperatures, the relation between environmental temperatures and flying insect abundance was first assessed. This was then used to objectively determine the most meaningful critical temperatures for cold snap definition, and then how cold snap frequency and duration relate to patterns of chick mortality.

### Insect abundance

Starting in 1989, a 12-m Rothamsted suction sampler (Macaulay et al. 1988) located at the Cornell University Experimental Ponds, Unit 1, has been run to collect daily insect samples during each swallow field season (except 1996), from the beginning of April to, on average, the end of July. Research in the United Kingdom (Taylor 1963; Macaulay et al. 1988) and in Ithaca (McCarty 1995) has demonstrated that a sampler 12 m in height produces insect samples that are well homogenized horizontally to avoid the effects of changing and variable terrestrial habitat distributions, providing a standardized regional assessment of the general abundance of flying insects available on a given day.

The sampler generally ran from 0700 to 1800 hours every day, and the actual time on was recorded every day, with numbers standardized to insects captured per hour of sampler operation. Insect samples were cleaned and transferred from collection jars into storage vials filled with 70 % ethanol. Samples were then identified to taxonomic order, sized, and counted, though the results here are based on total numbers of all insects except thrips (Thysanoptera) in the samples.

### Temperature

Ithaca weather data were provided by the Northeast Regional Climate Center, which uses an Ithaca weather station located approximately 8 km south of our primary field site. Because we assumed that flying insects required a particular threshold temperature to fly (Taylor 1963), we used maximum temperatures rather than average daily temperatures as the most valuable index of operational temperature.

### Cold snaps

**Fig. 2:**
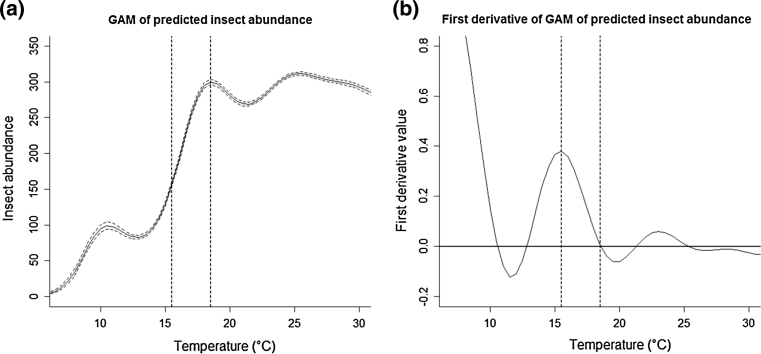
**a** Generalized additive model displaying the predicted flying insect abundance at a given maximum temperature (in °C). The model was constructed using all of the counted insect samples from 1989 to 2009 between May 17 and August 4 (*n* = 882), and the GAM prediction (*solid curve*) and its 95 % confidence interval (*dashed curve*) are shown. **b** The first derivative of (**a**), used to determine the threshold temperatures (*vertical dashed lines* in both figures)

To objectively define cold snaps independently from the chick mortality data, we explored the relationship of insect abundance to daily maximum temperature for the insects captured during daily breeding-season samples from 17 years between 1989 through 2009. To increase the relevance of insect abundance as a measure of food supply, we only used samples collected during the tree swallow nestling period (May 17–August 4), defined by the earliest recorded date of a chick hatching and the latest recorded date of a chick dying or fledging. We used all insects in these samples except thrips, as studies of this population (McCarty and Winkler 1999b) show that the only prey actively avoided are those less than 3 mm long. Since this relationship was not linear and the dependent variable (insect abundance) had a count nature, we used a generalized additive model with a logarithmic link function and a Poisson distribution for insect abundance (GAM, using function gam() in package mgcv in R 2.14.0; Wood 2011; R Development Core Team 2011). Using this model, we evaluated several candidate threshold temperatures, eventually choosing two based on the maximum derivative and abundance on the GAM curve (Fig. 2). All days with maximum temperatures lower than these critical values were considered to be in a cold snap. We also considered cold snaps of three different durations: one, two and three consecutive days below the threshold temperature.

### Chick mortality

Nest boxes designed for tree swallows were placed at numerous sites throughout Tompkins County, New York. At each field site, nest boxes were checked for the presence of nesting material, eggs, and chicks, beginning in mid-April, when birds have historically initiated nesting. These checks occurred at varying intervals, usually every 2 days. Field crews have been collecting data for each field season since 1986, and a long-term database derived from these checks contains data on nesting periods, clutch sizes, lay dates, hatching times, brood sizes, fledging times, and brood fates. Note that data on chick fates and dates have been recorded in this database at the whole brood level, and studies like the current one, needing to consider the fate-dates of individual chicks in a brood, must laboriously search through the original nest-check field books to reconstruct individual chick histories. To learn how changes in temperature and insect abundance may affect overall breeding success, the years of 1992, 2000, and 2006 were selected for such a detailed reconstruction based on their notably poor breeding success: distinctively, less than 60 % of attempted nests produced at least one fledgling in those years (Fig. 1). The year 2002 was also selected for analysis as a control year because of excellent fledgling production in that year. All broods subjected to experiments that would have affected their risk of mortality were excluded from further analysis. For each of these four field seasons analyzed, individual chick mortality histories were recovered from the original field records and used to test the hypothesis that cold snaps during the breeding season were linked to spikes in chick mortality. In total, 2,361 chicks from 554 nests were used in this more-focused “individual-level” study.

The largest complication in this analysis was that the exact date of chick mortality could not be determined. Nest boxes were not checked every day, and each chick mortality date was associated with an accuracy code: the number of days since the last check that the chick was still alive. To compensate for this uncertainty in dates of chick mortality, for visualizations of patterns of chick mortality (and not statistical analyses) a chick was assumed to contribute a fraction of its fate towards each day that it could have died. For example, a chick that was found dead 2 days after it was last seen alive would contribute 1/2 deaths to the 2 days when it could have died. To determine the proportion of chicks that died compared to those at risk, we used an analogous fractional-death procedure to calculate how many nestlings were alive and at risk of dying on each of the days in the breeding seasons studied. Resulting graphs showing the time-course of cold snaps, insect abundance, numbers of chicks at risk, and numbers of chicks dying were used to visualize the general relationships among these variables.

The lack of precision in timing of death is what led us to test nest-survival models (Dinsmore et al. 2002) with procedures in the nest-survival module of MARK because they account for imprecision in dates of chick mortality. Although the nest-survival module of MARK can distinctively deal with the lack of temporal precision in our data, it has the compensating disadvantage that, as a saturated model, no estimates of model goodness-of-fit can be calculated (Rotella 2009). A *χ* ^2^ test of the independence of chick fates (Dinsmore and Knopf 2005) showed that chick fates within a brood were not independent (*χ*_46_^2^ = 25,627.71, *p* < 0.001). Thus, we based another set of models on measures of mortality at the brood level using three different definitions of brood mortality: those in which the death of all chicks, only half the chicks, or only one chick in a brood was required to qualify the brood as having experienced mortality.

**Fig. 3:**
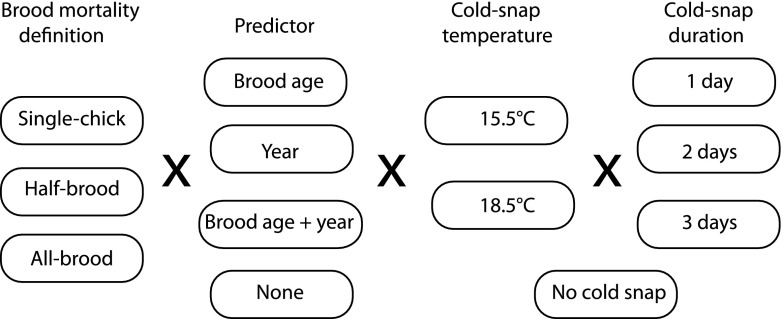
Schematic for the various models tested for their effects on brood survival. Each of the models tested involved one brood definition and at least one predictor and/or cold snap definition (consisting of a threshold temperature and a duration)

For both sets of analyses, we modeled the daily survival rates (DSRs) of broods using nest survival models in Program MARK (White and Burnham 1999) through the RMark interface from R (Laake and Rextad 2011). We tested all models with brood age (days since the hatching of the first chick in the brood), year (as a factor, not a continuous predictor), and the occurrence of cold snaps as predictors of chick mortality. Though we would normally test interactions among these predictor variables, we chose not to do so in this case because cold snaps, which have such a large effect on chick mortality, are so patchily distributed across years, especially in June when the largest effects on chick mortality occur (Supplementary material). Thus, given the sparse and sporadic nature of cold snaps and mortality, any test of cold snap interaction with year or brood age would be unreliable. Because we were interested in estimating the general relationship between cold snap occurrence and mortality and its overall interaction with brood age, not the differences in each of these across years, we concentrated on estimating each of these relationships without the interactions with year. Models with each of the candidate definitions of cold snaps were tested to determine the cold snap threshold and duration that best captured the effect on chick mortality (Fig. 3). All candidate models were compared using Akaike’s information criterion (AIC; Akaike 1974), and differences between models were assessed by the difference in AIC scores (ΔAIC) from the model with the smallest AIC in the set being compared.

At first, analyses were based on the focused dataset of 4 years that included detailed reconstructions of individual chick histories. Because these analyses demonstrated that chick fates depended on those of their brood mates (i.e., were not independent), we exploited a larger set of 11 years, many with much lower overall chick mortality rates, in which the quantity and quality of data was sufficient to explore the relation between temperatures and mortality. This expanded our analysis to 2,261 nests. These coupled analyses are defined as “individual” and “brood-level” analyses of mortality, respectively.

## Results

### Cold snaps

We found two critical temperature values from the GAM prediction for insect abundance with respect to maximum daily temperature (Fig. 2a) and its derivatives (Fig. 2b). The first threshold value was 15.5 °C. As the maximum of the first derivative, this value showed maximal rate of change of insect abundance per degree change in temperature. We defined the second threshold value as 18.5 °C, which corresponds to the local maximum of insect abundance with respect to temperature. These temperatures were used to classify whether a day was part of a 1-, 2-, or 3-day cold snap.

### Weather, insects and chick mortality–individual-level analysis

**Fig. 4:**
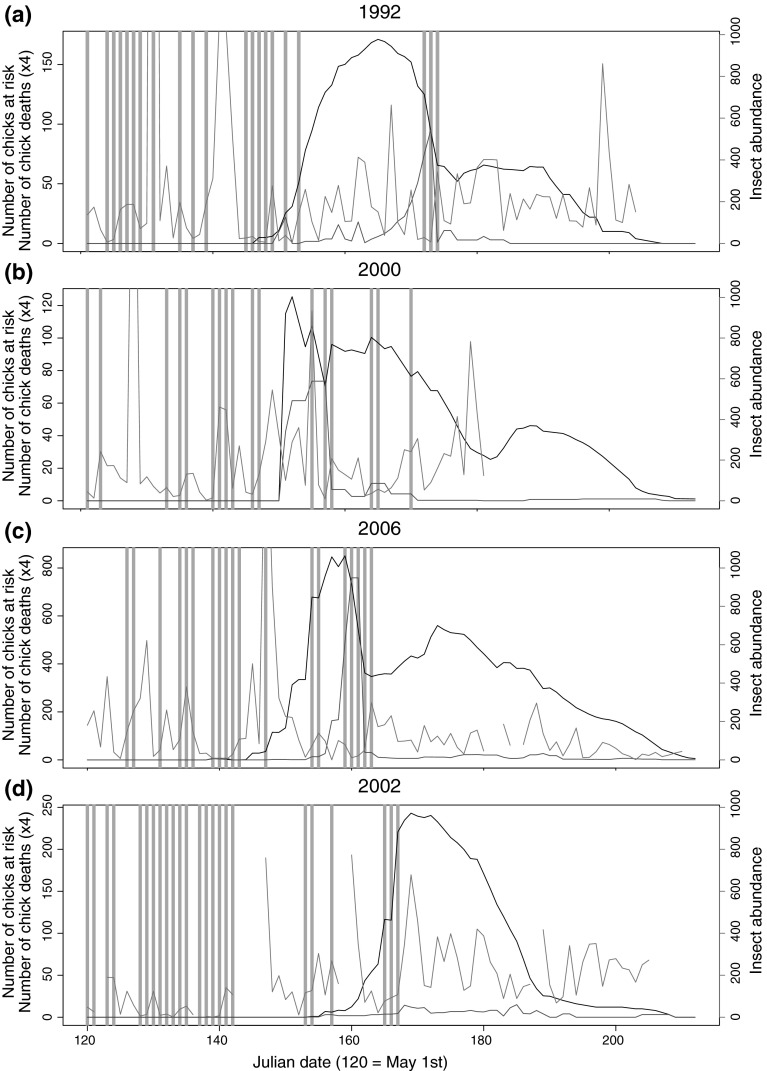
Combined graph of insect abundance (*purple*), number of chicks at risk of death on a given day (*black*), calculated number of chick deaths (*red*), and cold snap occurrence by Julian dates for: **a** 1992, **b** 2000, **c** 2006, and **d** 2002. *Light blue vertical bars* indicate days that were part of a cold snap as defined by the 18.5° maximum temperature threshold. Number of chicks dying each day is multiplied by four to allow plotting on the same *y* axis as chicks at risk (color figure online)

Each of the 3 years of high overall chick mortality (1992, 2000, and 2006) showed patterns of mortality that suggested an association between the occurrence of cold snaps and peaks in chick mortality (Fig. 4a–c), reinforced by the lack of cold snaps and spikes in chick mortality in the year of low overall chick mortality (2002; Fig. 4d). In all these years, cold snaps occurred in May and June, but they only had large effects on chick mortality when they occurred with large numbers of chicks at risk (Fig. 4). In some of these years, the spikes in chick mortality seemed to be shifted slightly earlier than the cold snap (e.g., 2000 and 2006; Fig. 4b, c), but this almost certainly resulted from the lack of precision in estimated time of death for the individual chicks in the brood.

Of the 27 models tested, the one with a year and brood age effect on chick mortality along with a 3-day 18.5 °C cold snap definition had the lowest AIC value (4,306.86; Table 1). The model with the next smallest AIC differed only in not having a year effect, but its AIC was 71.42 larger than the best model (Table 1). Cold snaps had a pervasive effect on chick mortality: the top 24 models tested all included cold snaps in the model for chick mortality (Table 1).

**Table 1:** AIC values for analyses based on detailed reconstructions of individual tree swallow (*Tachycineta bicolor*) chick histories (see text)

Model	Number of parameters	AIC	ΔAIC	Weight	Deviance
Year + brood age + 3 day 18.5°	6	4,306.86	0	1.00	4,294.86
Brood age + 3 day 18.5°	3	4,378.28	71.42	0.00	4,372.28
Year + 3 day 18.5°	5	4,428.49	121.63	0.00	4,418.48
3 day 18.5°	2	4,526.77	219.91	0.00	4,522.77
Year + brood age + 1 day 18.5°	6	4,540.69	233.83	0.00	4,528.69
Brood age + 1 day 18.5°	3	4,555.71	248.85	0.00	4,549.71
Year + brood age + 2 day 18.5°	6	4,592.86	286.00	0.00	4,580.86
Brood age + 2 day 18.5°	3	4,598.42	291.56	0.00	4,592.42
Year + 1 day 18.5°	5	4,836.34	529.48	0.00	4,826.34
Year + brood age + 1 day 15.5°	6	4,859.87	553.01	0.00	4,847.86
Year + 2 day 18.5°	5	4,860.10	553.24	0.00	4,850.09
1 day 18.5°	2	4,862.35	555.50	0.00	4,858.35
Year + brood age + 2 day 15.5°	6	4,887.25	580.39	0.00	4,875.24
2 day 18.5°	2	4,889.16	582.30	0.00	4,885.16
Brood age + 1 day 15.5°	3	4,901.13	594.27	0.00	4,895.13
Brood age + 2 day 15.5°	3	4,915.26	608.40	0.00	4,909.26
2 day 15.5°	2	5,245.68	939.82	0.00	5,242.68
1 day 15.5°	2	5,246.77	939.91	0.00	5,242.77
Year + 1 day 15.5°	5	5,248.62	941.76	0.00	5,238.62
Year + 2 day 15.5°	5	5,249.83	942.97	0.00	5,239.83
Year + brood age + 3 day 15.5°	6	5,811.12	1,504.26	0.00	5,799.12
Year + 3 day 15.5°	5	5,824.90	1,518.04	0.00	5,814.90
Brood age + 3 day 15.5°	3	5,959.76	1,652.90	0.00	5,953.76
3 day 15.5°	2	5,968.45	1,661.59	0.00	5,964.45
Year + brood age	5	6,023.54	1,716.68	0.00	6,013.54
Year	4	6,044.14	1,737.27	0.00	6,036.13
Brood age	2	6,085.60	1,778.74	0.00	6,081.60

### Weather, insects and brood mortality–brood-level analyses

Recall that the individual-level analyses were focused on the few key years of highest contrasts in chick mortality. The brood-level analysis of 11 years (including the 4 analyzed at the individual level) allowed us to look at a larger sample of years, which had intermediate levels of chick mortality. In this analysis, in addition to year, brood age, and the various definitions of cold snap durations and severities were retained from the individual-level analyses, and we analyzed a set of 27 models for each of the three criteria for brood mortality (Fig. 3), resulting in a total of 81 models. Of these, models with all-brood mortality (AIC range of the top 10 models: 2,748.4–2,867.9) explained much more of the variance than did models based on half-brood (AIC range of top 10: 3,777.4–3,959.0) and single-chick mortality (AIC range of top 10: 5,363.2–5,477.7). Among the all-brood mortality models (Table 2), a model using a cold snap definition of 18.5 °C and 1-day duration had the lowest AIC value, and the next best model, with a 2-day rather than 1-day cold snap definition at the same temperature was only slightly worse (ΔAIC = 2.2). Comparing the parameter estimates for these two models (Table 3) demonstrates a great similarity in the coefficients, their standard errors, and confidence limits for the effects of both brood age and cold snaps of both the 1-day and 2-day cold snap definitions. Though the top 2 models performed much better than all the others, the top 21 models all had cold snaps as an important source of brood mortality (Table 2). (The top 15 models using the half-brood mortality criterion and the top 11 models with the single-chick criterion also included cold snaps.)

**Table 2:** AIC values for analyses of larger sample of years based on whole-brood fate-dates and success (see text)

Model	Number of parameters	AIC	AAIC	Weight	Deviance
Year + brood age + 1 day 18.5°	13	2,748.36	0	0.75	2,722.35
Year + brood age + 2 day 18.5°	13	2,750.55	2.19	0.25	2,724.54
Brood age + 2 day 18.5°	3	2,798.53	50.17	0.00	2,792.53
Brood age + 1 day 18.5°	3	2,809.52	61.16	0.00	2,803.52
Year + brood age + 3 day 18.5°	13	2,810.81	62.46	0.00	2,784.80
Year + 2 day 18.5°	12	2,823.42	75.06	0.00	2,799.41
Year + 1 day 18.5°	12	2,826.78	78.42	0.00	2,802.77
Year + 3 day 18.5°	12	2,847.06	98.71	0.00	2,823.05
Year + brood age + 2 day 15.5°	13	2,857.75	109.39	0.00	2,831.74
Brood age + 3 day 18.5°	3	2,867.87	119.51	0.00	2,861.86
2 day 18.5°	2	2,868.60	120.24	0.00	2,864.60
Brood age + 2 day 15.5°	3	2,873.07	124.71	0.00	2,867.07
Year + brood age + 1 day 15.5°	13	2,877.36	129.00	0.00	2,851.34
1 day 18.5°	2	2,886.56	138.21	0.00	2,882.56
Year + brood age + 3 day 15.5°	13	2,898.37	150.02	0.00	2,872.36
3 day 18.5°	2	2,905.57	157.21	0.00	2,901.56
Year+ 2 day 15.5°	12	2,911.48	163.12	0.00	2,887.47
Brood age +1 day 15.5°	3	2,918.38	170.02	0.00	2,912.38
Year+ 3 day 15.5°	12	2,925.23	176.87	0.00	2,901.22
Year + 1 day 15.5°	12	2,930.88	182.52	0.00	2,906.87
2 day 15.5°	2	2,935.78	187.42	0.00	2,931.78
Year + brood age	12	2,939.19	190.83	0.00	2,915.18
Brood age + 3 day 15.5°	3	2,951.38	203.02	0.00	2,945.38
Year	11	2,968.01	219.66	0.00	2,946.00
Brood age	2	3,004.54	256.19	0.00	3,000.54
1 day 15.5°	2	3,494.08	745.73	0.00	3,490.08
3 day 15.5°	2	10,089.79	7,341.43	0.00	10,085.78

**Table 3:** Coefficient estimates for the top model in Table 1, with standard errors of the estimates and lower and upper 95 % confidence bounds

Source	Estimate	se	Lower confident limit	Upper confident limit
Intercept	5.42	0.15	5.11	5.72
Season 2000	−1.19	0.18	−1.54	−0.84
Season 2002	0.57	0.18	0.21	0.92
Season 2006	−0.17	0.12	−0.39	0.06
Brood age	−0.07	0.006	−0.09	−0.06
3-day 18.5°	−3.36	0.09	−3.52	−3.19

The AIC for this model was 4,306.86, and all predictor effects were modeled on the logit scale

Note that these brood-level analyses, while reaffirming the importance of cold snaps, differed slightly only in the cold snap duration. The 9 and 8 best models at the individual and brood levels, respectively, all included a cold snap threshold of 18.5°, but the 2 best models at the brood-level analysis included cold snap durations of 1 and 2 days instead of the three-day duration supported by the individual-level analysis.

## Discussion

Analyses at both scales indicated that the critical temperature for defining cold snap effects on chick mortality was 18.5°, a level that corresponds to the peak in the GAM of insect abundance on temperature and the start of a generally high level of insect availability over a wide range of permissive temperatures (Fig. 2). It is interesting that swallows thus seem to be suffering the effects of reduced prey availability as soon as insect availability begins to decline: they are apparently dependent on constant high levels of prey availability, and any deviation from this high plateau in food supply throws a switch to reduced parental care and, at least at the Ithaca sites, emigration from the upland breeding sites to more reliable, and more distant, foraging over large low bodies of water.

The 4 top models in the individual-level analysis of the years with greatest mortality all agreed on a 3-day cold snap duration (Table 1). This contrasts with the results of the brood-level analysis (Table 2), which favored a cold snap duration of 1 or 2 days in its top 4 models. This result accords with the impression over the years that high brood mortalities are associated with extended periods of cold weather (see, e.g., Fig. 4a, b). Though single-day cold snaps can have a large effect on chick mortality (Fig. 4c), the association of chick mortality with longer cold snap durations probably arises from the fact that the set of years for the individual-level analyses included the 3 years of highest recorded chick mortality in this population, and these years have both more days with longer cold snaps and more mortality.

Despite this slight difference in the effects of cold snap duration in the two datasets, the message of these analyses is clear: there is a strong link between temperatures cold enough to affect flying insect abundance, the number of chicks at risk, and chick daily survival rate. Given studies of other passerines showing the correlation between nestling growth and food supply, the combination of cold temperatures (and thus potentially higher metabolic demand) and low insect abundance likely have significant impacts, not only on chick mortality (Bryant 1973; Verboven et al. 2001; Both 2010) but also on chick growth and subsequent success, as more food is given to chicks when the food supply is abundant, and chicks often have difficulty recovering from periods of delayed growth (Jones 1988; McCarty 2001).

Statistical analyses confirmed the visual evidence (Fig. 4) that the brood DSR is significantly lower when temperatures are cold. With such a preponderance of the top models in all analyses including a cold snap effect on whole brood mortality, there is little doubt that cold snaps occurring during the nestling period cause spikes in chick mortality. The top models (Tables 3, 4) also included a negative brood age effect, which indicate a linear decrease in DSR with chick age. This could be a physiological result of the chicks’ development of thermoregulatory abilities, gradually shifting towards endothermy with higher energetic costs (Marsh 1980; McCarty 1995). Our general naturalist impression is that, when a cold snap hits, most of the parents on our nest-box grids disappear. One possible hypothesis to explain lower survival rates in older chicks may be that adults feeding older chicks are forced to spend more time away from the nest to meet the larger food demand of larger chicks, but we have no data on parental attendance during cold snaps to test this. We think it likely that the principal effect leading to decreasing survival under cold snaps with increasing chick age is that older chicks need more food, are more vulnerable to nutritional shortages, and have a harder time recovering from a period of thermal and nutritional stress. As consistent as this interpretation is with what we know of the biology of developing swallows, it is important to note that the ages in our data were too sparsely distributed to effectively test for non-linear effects of cold snaps on chick mortality, as we expect from previous research that mortality is likely to experience strong threshold effects with age. McCarty (1995) proposed that younger pre-homeothermic chicks had higher survival through periods of low temperatures because they did not struggle metabolically to retain body temperature and instead allowed their temperature to fall with ambient temperatures, saving costs both in thermogenesis and in reduced thermal gradient and heat loss. Thus, future research is still needed to better understand the functional form of the interaction between chick age and the effects of cold on survival.

**Table 4:** Coefficient estimates for the top two models in Table 2, with standard errors of the estimates and lower and upper 95 % confidence bounds

Source	Estimate	SE	Lower confidence limit	Upper confidence limit
Model a				
Intercept	5.16	0.27	4.64	5.68
Season 2000	0.32	0.34	−0.35	0.99
Season 2002	1.28	0.50	0.29	2.27
Season 2003	1.59	0.32	0.97	2.22
Season 2004	1.63	0.32	1.01	2.26
Season 2005	1.39	0.26	0.84	1.95
Season 2006	0.62	0.25	0.13	1.11
Season 2007	0.77	0.28	0.21	1.33
Season 2008	1.21	0.31	0.60	1.83
Season 2009	1.19	0.32	0.57	1.82
Season 2010	0.33	0.25	−0.17	0.33
Brood age	−0.09	0.01	−0.11	−0.07
1-day 18.5°	−2.18	0.14	−2.45	−1.90
Model b				
Intercept	5.13	0.27	4.60	5.67
Season 2000	0.19	0.34	−0.48	0.87
Season 2002	1.33	0.50	0.34	2.31
Season 2003	1.44	0.32	0.81	2.06
Season 2004	1.41	0.32	0.79	2.04
Season 2005	1.45	0.28	0.90	2.01
Season 2006	0.69	0.25	0.19	1.19
Season 2007	0.54	0.29	−0.03	1.11
Season 2008	0.86	0.32	0.24	1.49
Season 2009	1.10	0.32	0.47	1.73
Season 2010	0.36	0.26	−0.14	0.87
Brood age	−0.09	0.01	−0.10	−0.07
2-day 18.5°	−2.28	0.15	−2.57	−1.99

The AIC for (*a*) was 2,748.36 and for (*b*) was 2,750.55, and all predictor effects were modeled on the logit scale

Overall, cold temperatures clearly have a significant effect on the survival of tree swallow broods, and, consequently, on the overall success of tree swallow breeding seasons. There are interesting biological complexities yet to be uncovered in both the precise causes of mortality and the role age plays in the changes of DSR. Further understanding of these connections between weather, avian physiology, and reproduction will contribute to a better understanding of the numerous potential impacts of temperature on the survival of tree swallows, as well as the impacts of global phenomena such as climate change on the health and conservation of all swallows and the ecosystems they live in.

## Electronic supplementary material

Below is the link to the electronic supplementary material.

Supplementary material 1 (DOC 222 kb)
