# Supplementary material for: Temperature effects on food supply and chick mortality in tree swallows (Tachycineta bicolor)
Source: Oecologia. 2013 Mar 7;173(1):129–38. doi: 10.1007/s00442-013-2605-z (PMC3751296; doi:10.1007/s00442-013-2605-z)

Supplementary material 1. The numbers of cold snaps occurring in each of the months of the swallow breeding season in Ithaca for years analyzed in this paper. Cold snaps are defined as days not having maximum temperatures in excess of 15.5° or 18.5° and durations of from one to three days.


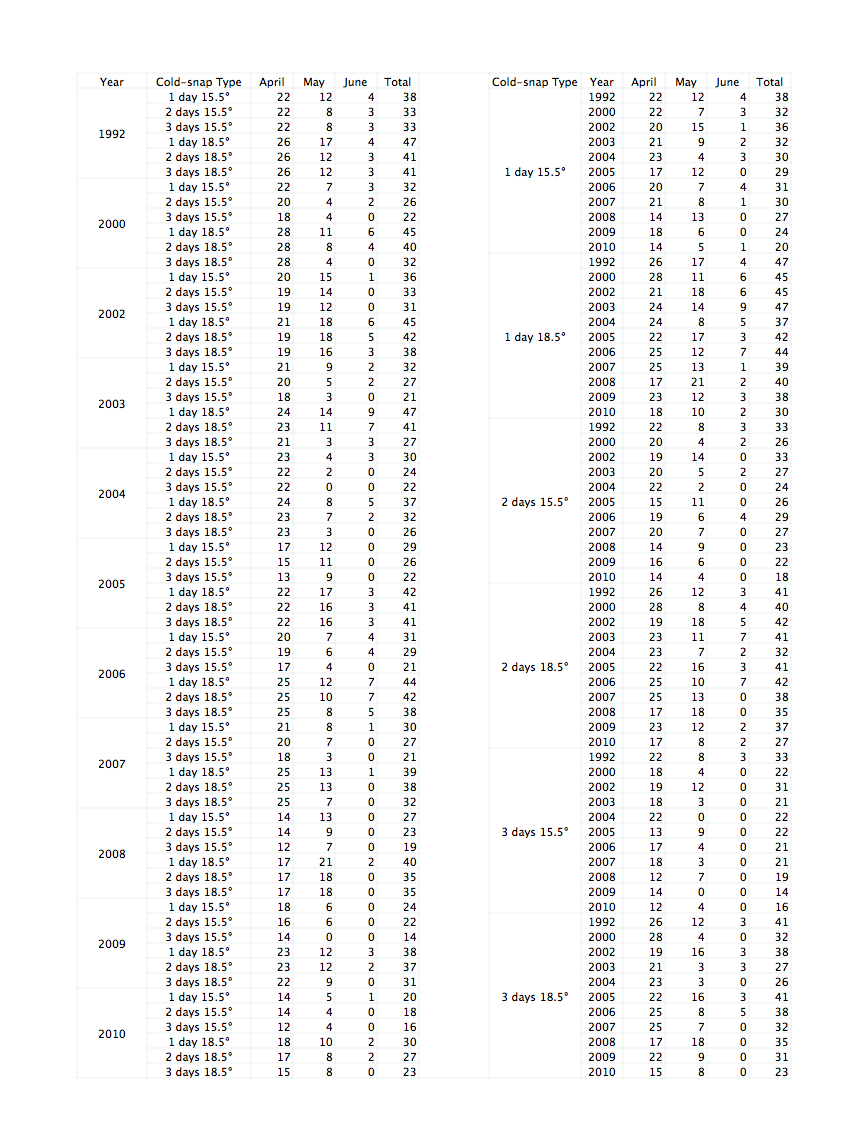

Supplement: Supplementary file 1 — Supplementary material 1 (DOC 222 kb) [file 442_2013_2605_MOESM1_ESM.doc]
